# Abnormal Whole Brain Functional Connectivity Pattern Homogeneity and Couplings in Migraine Without Aura

**DOI:** 10.3389/fnhum.2020.619839

**Published:** 2020-12-11

**Authors:** Yingxia Zhang, Hong Chen, Min Zeng, Junwei He, Guiqiang Qi, Shaojin Zhang, Rongbo Liu

**Affiliations:** ^1^Department of Radiology, West China Hospital of Sichuan University, Chengdu, China; ^2^Department of Radiology, The Third Affiliated Hospital of Chengdu Medical College, Pidu District People's Hospital, Chengdu, China

**Keywords:** migraine without aura, FCHo, functional connectivity, resting-state, functional magnetic resonance imaging

## Abstract

Previous studies have reported abnormal amplitude of low-frequency fluctuation and regional homogeneity in patients with migraine without aura using resting-state functional magnetic resonance imaging. However, how whole brain functional connectivity pattern homogeneity and its corresponding functional connectivity changes in patients with migraine without aura is unknown. In the current study, we employed a recently developed whole brain functional connectivity homogeneity (FcHo) method to identify the voxel-wise changes of functional connectivity patterns in 21 patients with migraine without aura and 21 gender and age matched healthy controls. Moreover, resting-state functional connectivity analysis was used to reveal the changes of corresponding functional connectivities. FcHo analyses identified significantly decreased FcHo values in the posterior cingulate cortex (PCC), thalamus (THA), and left anterior insula (AI) in patients with migraine without aura compared to healthy controls. Functional connectivity analyses further found decreased functional connectivities between PCC and medial prefrontal cortex (MPFC), between AI and anterior cingulate cortex, and between THA and left precentral gyrus (PCG). The functional connectivities between THA and PCG were negatively correlated with pain intensity. Our findings indicated that whole brain FcHo and connectivity abnormalities of these regions may be associated with functional impairments in pain processing in patients with migraine without aura.

## Introduction

Migraine is a common chronic and idiopathic disorder characterized by recurrent moderate to severe headaches (Kruit et al., 2004). Migraine is an important healthcare and social problem affecting the patients' quality of life. Moreover, frequent migraine attacks cause loss of pain, sensitivity and functional lesions in brain regions (Tietjen, 2004; Borsook et al., 2012). With structural and diffusion magnetic resonance imaging (MRI) techniques, a few previous studies have demonstrated that migraine results in reduced gray matter volume and disrupted white matter microstructural integrity (DaSilva et al., 2007; Kim et al., 2008; Schmidt-Wilcke et al., 2008; Valfrè et al., 2008). Based on resting-state functional MRI (fMRI), abnormal amplitudes of low-frequency fluctuation and regional homogeneity (ReHo) were also reported (Yu et al., 2012; Xue et al., 2013). All these studies demonstrated that migraine leads to progressive changes of brain structures and functions in patients.

Resting-state fMRI mainly reflects spontaneous fluctuations and has been widely used to study the intrinsic functional abnormalities in diseases (Fox et al., 2006; Yeo et al., 2011; Wang et al., 2017, 2020b). To characterize the functional activity similarities, ReHo was proposed to describe the similarity of time series (Zang et al., 2004). Given that more and more studies have demonstrated brain functions determined by its connectivity patterns with other brain areas (Passingham et al., 2002; Wang et al., 2015, 2019a), thus, to quantitatively characterize the voxel-wise similarity of whole brain functional connectivity pattern is vital to better reveal the functional abnormalities in brain. Recently, Wang et al. (2018a) developed a whole brain functional connectivity homogeneity (FcHo) method to delineate the voxel-wise whole brain functional connectivity pattern similarity. FcHo is defined by measuring the similarity of the whole brain functional connectivity pattern of a specific voxel with that of its nearest 26 neighborhood voxels. Compared to the ReHo approach, FcHo can better identify the association cortex areas with higher FcHo values than primary cortical areas, but for ReHo distribution, some primary cortical areas also represent high ReHo values (Wang et al., 2018a). Compared to functional connectivity density (FCD) and functional connectivity strength (FCS) methods, FcHo directly measures the similarity of the whole brain functional connectivity map of a given voxel with the nearest neighborhood voxels instead of counting the number of connectivities above a specific threshold (Wang et al., 2019b). Recently, the FcHo method has been used to explore the functional abnormalities in depression and its neuromodulation treatment mechanism (Wang et al., 2019b, 2020a). The FcHo method provides a new approach to exactly localize the functional abnormality in brain disorders.

In the current study, a fully data-driven FcHo method without any hypothesis was applied to reveal the abnormal functional connectivity patterns in 21 patients with migraine without aura and 21 gender and age matched healthy controls using resting-state fMRI data. Resting-state functional connectivity of the brain areas with changed FcHo was also performed to further reveal the corresponding disrupted functional networks in patients with migraine without aura.

## Materials and Methods

### Subjects

Twenty-one patients with migraine without aura (16 female and five male; mean age = 31.19 ± 6.38 years) and 21 healthy controls (13 female and eight male; mean age = 30.19 ± 6.3 years) were recruited at the Third Affiliated Hospital of Chengdu Medical College, Pidu District People's Hospital. All participants were right-handed. The diagnosis of migraine without aura was made according to the International Headache Society criteria. The inclusion criteria for patients with migraine without aura were follows: (1) Must not have suffered from a migraine attack for at least 72 h before experiment; (2) Must not have a migraine precipitated during or on the day after the scan; (3) for migraine and healthy controls, no lifetime history of head trauma, seizures, serious medical or surgical illness, substance abuse or dependence, or contraindications for MRI. The participants were excluded if structural abnormalities were detected on MRI examination. Written informed consent was provided and obtained from all the subjects. This study was approved by the local ethics committees of the Third Affiliated Hospital of Chengdu Medical College, Pidu District People's Hospital.

### Resting-State fMRI Data Acquisition

MRI data was acquired on a 3-Tesla Siemens MRI scanner in the Department of Radiology, the Pidu District People's Hospital of the third Affiliated Hospital of Chengdu Medical College, Chengdu, China. Foam padding and earplugs were used to reduce head motion and scanner noise. The participants were instructed to close their eyes and not fall asleep during scanning. Resting-state fMRI images were acquired using a gradient-echo echo-planar imaging (GRE-EPI) sequence with parameters: repetition time (TR) = 2000 ms, echo time (TE) = 30 ms, flip angle (FA) = 90^0^, matrix = 64 × 64, field of view (FOV) = 220 × 220 mm, slice thickness = 4 mm with inter-slice gap = 0.6 mm, 32 axial slices, and 250 time points.

### Resting-State fMRI Data Pre-processing

The resting-state fMRI data was pre-processed using DPARSFA software (https://www.nitrc.org/projects/dparsf/). The preprocessing steps include: discarding the first 10 volumes to facilitate magnetization equilibrium; after slice timing, the remaining images realigned to the first volume to correct head motion; normalizing all the images to the EPI template in MNI space and resampled to a 3 × 3 × 3mm^3^; regression of Friston 24-parameter model of head motion, white matter, cerebrospinal fluid, and global mean signals and filtered with a temporal band-pass of 0.01–0.1 Hz. To exclude the head motion effects, the subjects with head-movement that exceeded 3 mm of translation or 3° of rotation in any direction were discarded. Under this criterion, no subject was discarded. Moreover, “scrubbed” was performed to censor the bad images before 2 time points and after 1 time points exceeding the pre-set criteria (frame displacement: FD, FD < 0.5) (Power et al., 2012). For the following functional connectivity analyses, the fMRI data were normalized to the EPI template and smoothed using a Gaussian kernel of 6 mm full-width at half maximum (FWHM).

### Whole Brain Voxel-Wise FcHo Analyses

FcHo, measured using Kendall's coefficient concordance (KCC) (Kendall and Gibbons, 1990), was calculated for each voxel to describe the whole brain connectivity pattern similarity. The FcHo of a given voxel was defined by computing a KCC value of the whole brain functional connectivity of this voxel with those of its nearest 26 neighbors (see the following formula). Finally, a whole FcHo map for each subject was obtained and smoothed with 6 mm FWHM for statistical analyses. To determine the FcHo differences between patients with migraine and healthy controls, a two-sample *t*-test was performed to compare the FcHo maps between the two groups. The significance was determined using the Alphasim correction method in the DPARBI toolkit (http://rfmri.org/dpabi) with a threshold of *p* < 0.05 (cluster-forming threshold at voxel-level *p* < 0.001, cluster size 53).

$$\begin{array}{l} KCC=\frac{\sum {\left(R_{i}\right)}^{2}-K{\left(\bar{R}\right)}^{2}}{\frac{1}{12}N^{2}\left(K^{3}-K\right)} \end{array}$$

Where: *R*_*i*_ is the sum rank of the i th voxel of the whole brain; $\bar{R}=$ [(K+1)×N]/2 is the mean of the *R*_*i*_; *N* is the number of a given voxel and its nearest neighbors (*N* = 26); *K* is the number of whole brain voxels.

### Functional Connectivity Analyses

Seed-based whole brain functional connectivity analysis was used to further identify changed resting-state functional connectivity of the brain areas with different FcHo between patients with migraine and healthy controls. Functional connectivity was measured using Pearson's correlation coefficient between the averaged time series of the seed and target brain areas. Next, the Fisher's z transformation was applied to change functional connectivity to *Z* value to improve normality. The two-sample *t*-test was performed to identify brain areas with significantly different functional connectivity between patients with migraine without aura and healthy controls. The significance in the present study was determined using the Alphasim correction method in the DPARBI toolkit with a threshold of *p* < 0.05 (cluster-forming threshold at voxel-level *p* < 0.001, cluster size 47).

### Correlation Analyses

To determine the relationship between clinical assessments including visual analog scale (VAS), duration of illness and FcHo, and functional connectivities in brain areas showing differences between patients with migraine without aura and healthy controls, Spearman correlation analyses were performed, and the significance was set at *p* < 0.05.

## Results

### Demographics and Clinical Characteristics

The demographics and clinical characteristics of the subjects are shown in Table 1. No significant differences in gender (*p* = 0.51) and age (*p* = 0.61) were observed between patients with migraine without aura and healthy controls.

**Table 1:** Demographics and clinical characteristics of the subjects used in the present study.

	**Migraine**	**Controls**	***p-*value**
	**(*n* = 21)**	**(*n* = 21)**	
Gender (male/female)	16/5	13/8	0.51
Age (mean ± SD)	31.19 ± 6.38	30.19 ± 6.3	0.61
VAS (mean ± SD)	4.33 ± 1.46		
HAMD (mean ± SD)	6.71 ± 6.25		
HAMA (mean ± SD)	7.95 ± 6.77		
Duration of illness (months)	44.69 ± 61.13		
FD values	0.16 ± 0.29	0.15 ± 0.027	0.49

*A Pearson chi-squared test was used for gender comparison. Two-sample t-tests were used for age comparisons*.

*VAS, visual analog scale; HAMD, Hamilton Depression Rating Scale; HAMA, Hamilton Anxiety Scale*.

### Changed FcHo in MDD

**Figure 1:**
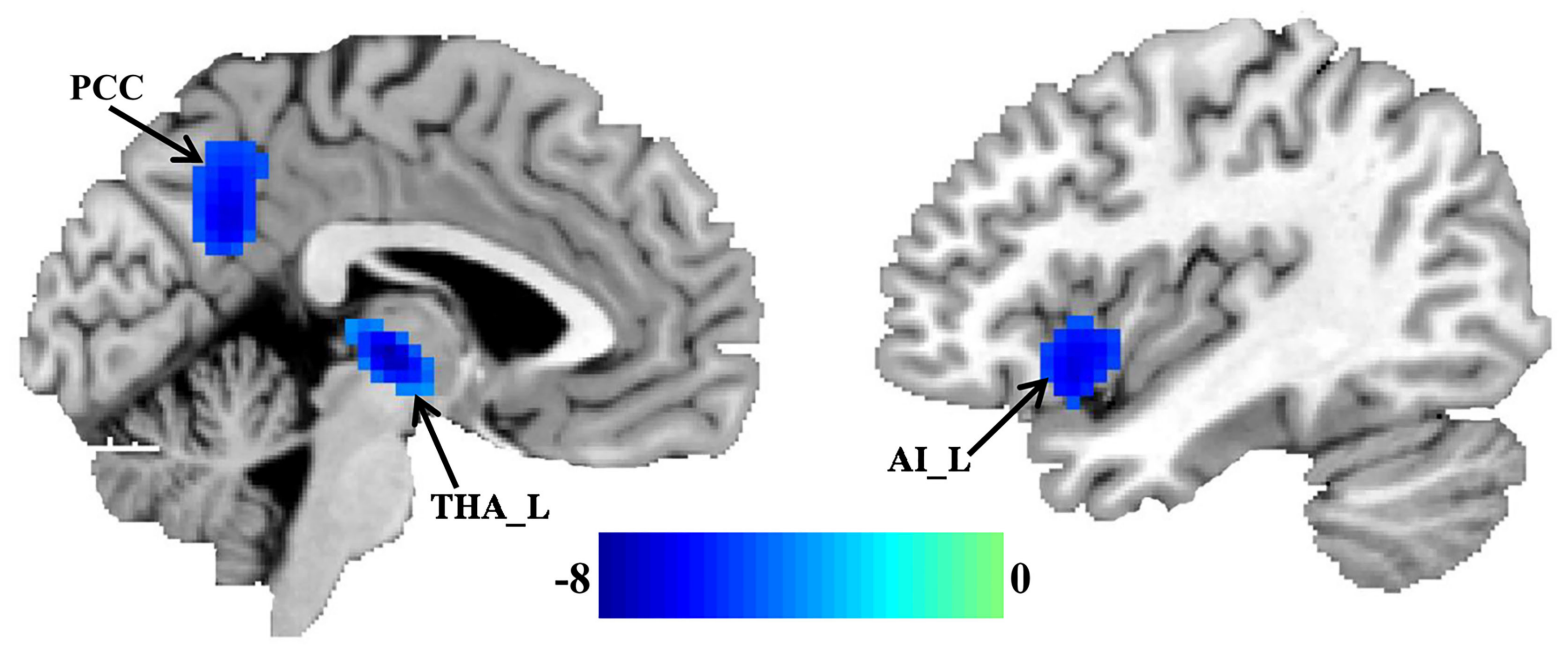
Decreased whole brain functional connectivity pattern homogeneity (FcHo) in patients with migraine without aura. Two-sample *t*-test was used to compare the FcHo maps between healthy controls and patients with migraine without aura and identified significantly decreased FcHo in the posterior cingulate cortex (PCC), thalamus (THA), and left anterior insula (AI) in patients with migraine without aura.

Significantly reduced FcHos in the posterior cingulate cortex (PCC), thalamus (THA), and left anterior insula (AI) were found in patients with migraine without aura compared with healthy controls (Figure 1 and Table 2).

**Table 2:** Regions with changed FcHo and functional connectivities in migraine patients.

**Parameters**	**Brain regions**	**Peak MNI coordinates**			***T* values**	**Cluster size**
		**X**	**Y**	**Z**		
FcHo:	Anterior insula	−45	15	−12	−7.61	143
	Posterior cingulate cortex	0	−54	30	−6.87	129
	Thalamus	−3	−18	0	−7.12	159
FC:	Anterior cingulate cortex	0	27	27	−7.28	126
	Medial prefrontal cortex	0	57	15	−7.76	106
	Precentral gyrus	30	−3	57	−6.93	75

*FcHo, whole brain functional connectivity pattern homogeneity; FC, functional connectivity; MNI, Montreal Neurological Institute; L, left hemisphere; R, right hemisphere*.

### Changed Functional Connectivity

**Figure 2:**
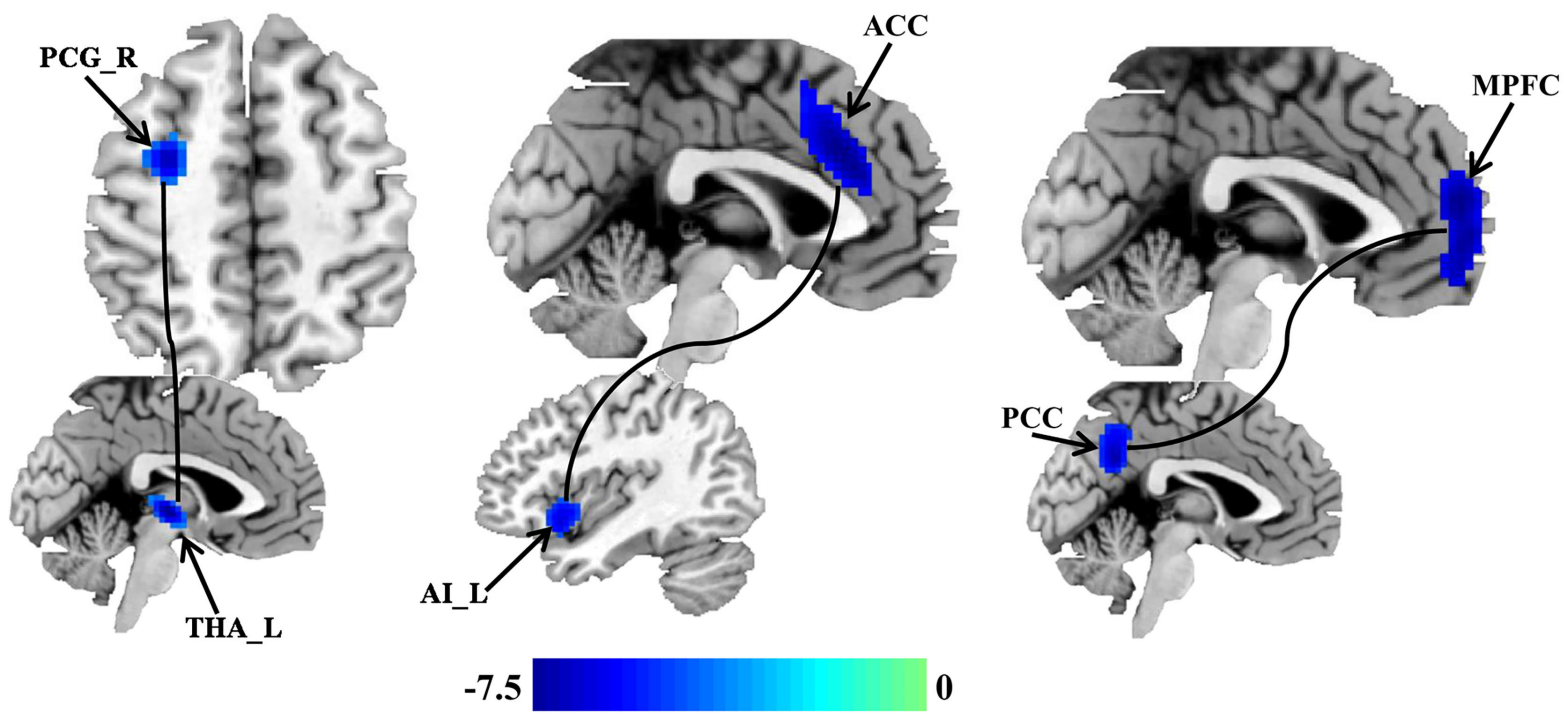
Disrupted resting-state functional connectivities in patients with migraine without aura. Seed based whole brain resting-state functional connectivities analyses of PCC, THA, and AI were performed, and two-sample *t*-tests were used to identify the significant differences in functional connectivities between patients with migraine without aura and healthy control groups. Seed-based functional connectivity analyses identified disrupted functional connection between the THA and left precentral gyrus (PCG), between the AI and anterior cingulate cortex (ACC), and between the PCC and medial prefrontal cortex (MPFC) in patients with migraine without aura.

Seed-based whole brain functional connectivity analyses were performed and decreased functional connection was identified between THA and left precentral gyrus (PCG), between AI and anterior cingulate cortex (ACC), and between PCC and medial prefrontal cortex (MPFC) in patients with migraine without aura compared with healthy controls (Figure 2 and Table 2).

### Correlation Analyses

**Figure 3:**
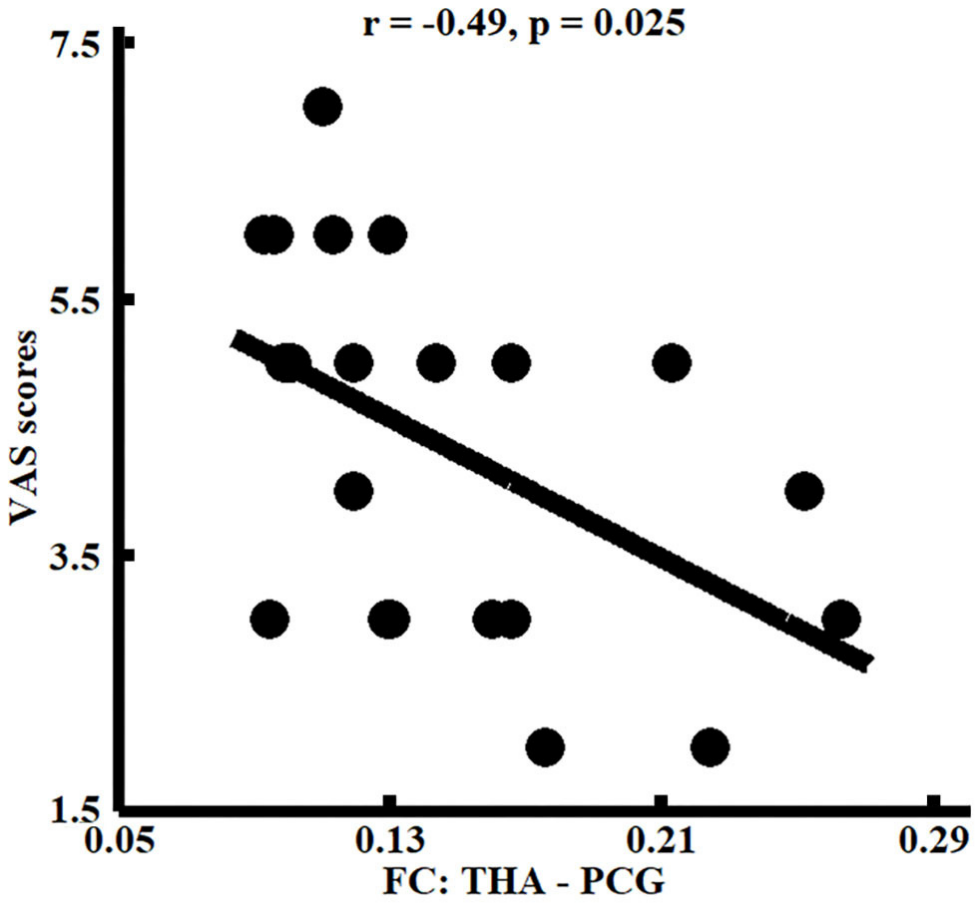
Correlation analyses between whole brain functional connectivity homogeneity (FcHo) and functional connectivities parameters and clinical performances. Significant correlation between the functional connectivities of THA with PCG and pain intensity (i.e., VAS scores) was identified in patients with migraine without aura.

Correlation analyses found that functional connectivities of THA with left PCG were significantly negatively correlated with the VAS scores (*r* = −0.49, *p* < 0.025) in patients with migraine without aura (Figure 3).

## Discussion

In the present study, whole brain functional connectivity patterns homogeneity (FcHo) and functional connectivity were used to localize functionally abnormal brain areas and their corresponding circuits in patients with migraine without aura. FcHo analysis revealed significantly decreased FcHo in PCC, AI, and THA in migraine without aura patients. Resting-state functional connectivity analysis further identified decreased functional connectivity between PCC and MPFC, between AI and ACC, and between THA and left PCG in patients with migraine without aura. Moreover, the changed functional connectivities of THA with left PCG were negatively associated with pain intensity. Our findings provide the neuroanatomical basis for migraine without aura and could help improve our understanding of the neuropathology of migraine.

Thalamus, as a relay hub, participates in the transmission of nociceptive inputs to the cortical areas (Kupers and Kehlet, 2006). The abnormal functional activities in thalamus during spontaneous attacks of migraine have been reported in previous studies (Kobari et al., 1989; Kupers and Kehlet, 2006). Thalamus and precentral gyrus are two important nodes in sensorimotor network which plays a key role in pain intensity processing and discrimination of the sensory components of pain perception (Kanda et al., 2000; Peyron et al., 2000). The correlation analysis in our study found that the functional connectivities between thalamus and precentral gyrus were closely related to pain intensity, suggesting the important role of sensorimotor network in pain perception and pain intensity coding. Thus, the decreased whole brain functional connectivity pattern similarity, and disrupted functional connections between the thalamus and precentral gyrus, indicated the abnormal pain perception and sensation processing in patients with migraine without aura.

Decreased whole brain functional connectivity pattern similarity in posterior cingulate cortex (PCC) and functional connections between PCC and medial prefrontal cortex (MPFC) were also found in patients with migraine without aura. PCC and MPFC are two core regions of default mode network (DMN), and have been reported to be associated with social cognition, emotional, and self-referential processing (Gusnard et al., 2001; Amodio and Frith, 2006; Etkin et al., 2011; Wang et al., 2019a). The abnormal functional activities in DMN found in our study were supported by previous studies which found changed local regional homogeneity (ReHo) and amplitude of low frequency fluctuations (ALFF) in patients with migraine without aura (Yu et al., 2012; Xue et al., 2013). Xue et al. (2013) found decreased ALFF values in the left rostral anterior cingulate cortex, bilateral prefrontal cortex, and increased ALFF values in the right thalamus. Yu et al. (2012) found decreased ReHo values in the right rostral anterior cingulate cortex, the prefrontal cortex, the orbitofrontal cortex, and the supplementary motor area. The abnormal prefrontal cortex and anterior cingulate cortex were consistently found in our study. The different change patterns may result from different analytical methods suggesting different methods could provide Supplementary Material. In addition, Xue et al. (2013) found increased ALFF in thalamus but we found decreased FcHo in this area. The inconsistency may be related to different analytical methods which reflect different functional information of this area.

In addition, decreased whole brain functional connectivity pattern similarity in anterior insula (AI) and functional connections between AI and dorsal anterior cingulate cortex (ACC) were identified in patients with migraine without aura. Our finding was consistent with the previous reports which also found structural and functional abnormalities in insula and ACC (Peyron et al., 2000; Kim et al., 2008; Valfrè et al., 2008). Insula plays a key role in integrating multimodal information including cognition, emotion, pain and homeostasis (Kong et al., 2006; Craig, 2011; Kelly et al., 2012; Simmons et al., 2013). ACC mainly integrates the positive and negative affective responses of pain (Vogt, 2005; Shackman et al., 2011). AI and ACC are important parts of salience network for accommodating the dynamic interaction between the internal self-perception and external orient stimulus (Menon and Uddin, 2010; Menon, 2011). ACC mainly includes the rostral rACC and dorsal dACC, and rACC is manly involved in endogenous pain control through the endogenous opioid systems (Petrovic et al., 2002; Wager et al., 2004), whereas dACC is primarily involved in motor control and cognitive processing, especially attention (Touroutoglou et al., 2012). Therefore, the abnormal FcHo in AI and functional couplings between AI and dACC suggest impaired cognitive processing or motor control in patients with migraine without aura.

Global signal regression (GSR) is a controversial problem in fMRI study. Some studies considered that GSR could increase the negative correlation and exaggerate the between-group differences (Gotts et al., 2013; Saad et al., 2013), but a recent study demonstrated that GSR could better exclude head motion effects than no GSR for functional connectivity analyses (Satterthwaite et al., 2019). In our study, we found the similar results using the fMRI data with and without GSR, which suggests that our findings are robust and the intrinsic functional abnormalities in migraine.

The previous studies have demonstrated that chronic pain patients showed increased high-frequency BOLD oscillations (0.12–0.20 Hz) in the medial prefrontal cortex and parts of default mode network (Baliki et al., 2011). Recently, frequency-dependent bilateral anterior insular networks were identified (Wang et al., 2018b, 2020c). In our study, decreased FcHo and functional connectivities of default mode network in the frequency band of 0.01–0.1 Hz were found suggesting that the functional abnormalities of the default mode network are present in both low and high frequencies. Additionally, we also found abnormal FcHo and functional connectivities in sensorimotor and salience networks in the frequency band of 0.01–0.1 Hz. Given the frequency-dependent functional circuits of salience network (Wang et al., 2018b, 2020c), whether the differences in the two networks are frequency-specific needs to be further investigated in future studies.

There are some limitations in our study. First, we only recruited a small sample size of patients with migraine without aura, and a large dataset is necessary to provide more convincing evidence. Second, all the analyses in the current study were performed only in the band of 0.01–0.1 Hz. Given that the pain related effect may be located in the frequency band >0.1 Hz, band-limited or coherence methods may be used in our future study to further reveal the pain effects.

## Conclusion

In conclusion, this study revealed abnormal whole brain functional connectivity pattern homogeneity in THA, PCC, and AI and abnormal resting-state functional connectivities between THA and PCG, between PCC and MPFC, and between AI and ACC patients with migraine without aura. These findings indicate abnormal emption, pain, and cognitive processing in patients with migraine without aura. The dysfunctions of these areas may contribute to the onset of migraine in patients.

## Data Availability Statement

The raw data supporting the conclusions of this article will be made available by the authors, without undue reservation.

## Ethics Statement

The studies involving human participants were reviewed and approved by the Third Affiliated Hospital of Chengdu Medical College, Pidu District People's Hospital. The patients/participants provided their written informed consent to participate in this study.

## Author Contributions

All authors listed have made a substantial, direct and intellectual contribution to the work, and approved it for publication.

## Conflict of Interest

The authors declare that the research was conducted in the absence of any commercial or financial relationships that could be construed as a potential conflict of interest.
