# Supplementary material for: Abnormal Whole Brain Functional Connectivity Pattern Homogeneity and Couplings in Migraine Without Aura
Source: Front Hum Neurosci. 2020 Dec 11;14:619839. doi: 10.3389/fnhum.2020.619839 (PMC7759668; doi:10.3389/fnhum.2020.619839)

Figure S1. Decreased whole brain functional connectivity pattern homogeneity (FcHo) in patients with migraine without aura identified using fMRI without global signals regression. Patients with migraine without aura also showed significantly decreased FcHo in posterior cingulate cortex, thalamus, and left anterior insula compared to healthy controls.

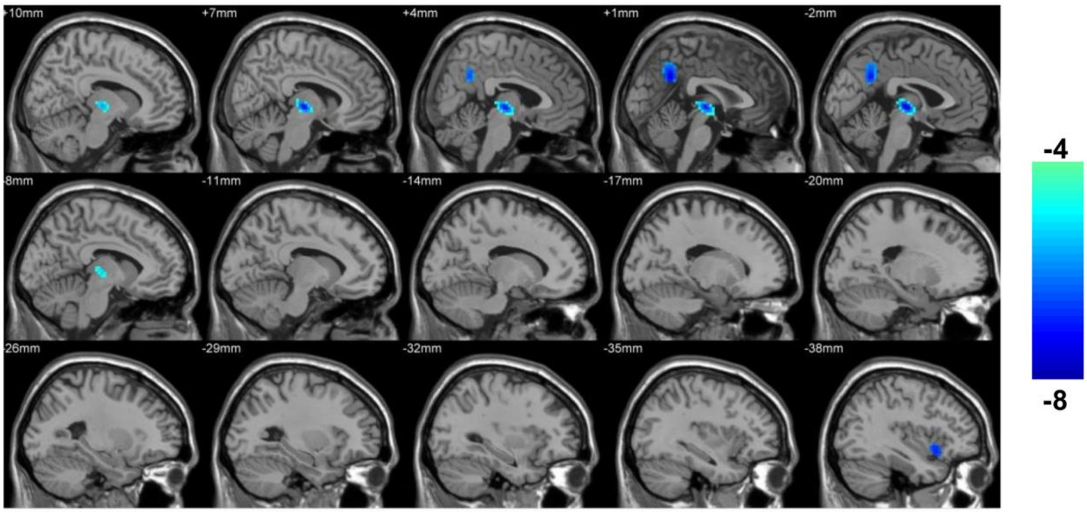

Figure S2. Decreased resting-state functional connectivities between thalamus and left precentral gyrus, between anterior insula and anterior cingulate cortex, and between posterior cingulate cortex and medial prefrontal cortex in patients with migraine without aura were found using fMRI data without global signal regression.

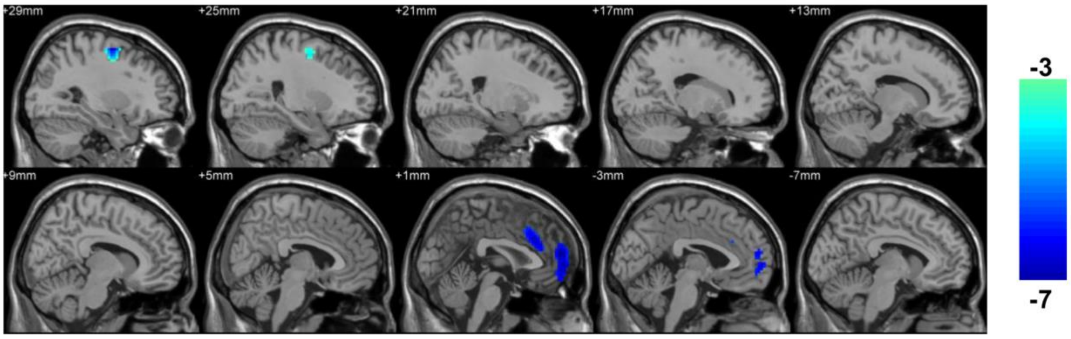

Supplement: Supplementary file 1 [file Data_Sheet_1.pdf]
